# Identification of differentially expressed genes regulated by transcription factors in glioblastomas by bioinformatics analysis

**DOI:** 10.3892/mmr.2014.3094

**Published:** 2014-12-15

**Authors:** BO WEI, LE WANG, CHAO DU, GUOZHANG HU, LINA WANG, YING JIN, DALIANG KONG

**Affiliations:** 1 Department of Neurosurgery, China-Japan Union Hospital of Jilin University, Changchun, Jilin 130033, P.R. China; 2 Department of Ophthalmology, The First Hospital of Jilin University, Changchun, Jilin 130021, P.R. China; 3 Department of Neurology, Jilin Oil Field General Hospital, Songyuan, Jilin 131200, P.R. China

**Keywords:** glioblastoma, differentially expressed gene, function enrichment analysis, weighted regulatory network, trimmed subnet

## Abstract

The present study aimed to identify differentially expressed genes (DEGs) regulated by transcription factors (TFs) in glioblastoma, by conducting a bioinformatics analysis. The results of the present study may provide potential therapeutic targets that are involved in the development of glioblastoma. The GSE4290 raw data set was downloaded from the Gene Expression Omnibus database, and consisted of 23 non-tumor samples and 77 glioblastoma (grade 4) tumor samples. Robust Multichip Averaging was used to identify DEGs between the glioblastoma and non-tumor samples. Functional enrichment analysis of the DEGs was also performed. Based on the TRANSFAC^®^ database, TFs associated with the glioblastoma gene expression profile were used to construct a regulatory network. Furthermore, trimmed subnets were identified according to calculated Z-scores. A total of 676 DEGs were identified, of which 190 were upregulated and 496 were downregulated. Gene Ontology analysis demonstrated that the majority of these DEGs were functionally enriched in synaptic transmission, regulation of vesicle-mediated transport and ion-gated channel activity. In addition, the enriched Kyoto Encyclopedia of Genes and Genomes pathway included neuroactive ligand-receptor interaction, calcium signaling pathway, p53 signaling pathway and cell cycle. Based on the TRANSFAC^®^ database, transcriptional regulatory networks with 2,246 nodes and 4,515 regulatory pairs were constructed. According to the Z-scores, the following candidate TFs were identified: TP53, SP1, JUN, STAT3 and SPI1; alongside their downstream DEGs. TP53 was the only differentially expressed TF. These candidate TFs and their downstream DEGs may have important roles in the progression of glioblastoma, and could be potential biomarkers for clinical treatment.

## Introduction

Glioblastoma is the most frequent and aggressive brain malignancy in adults, and is characterized by a heterogeneous population of cells that are involved with progression of the disease (1). It is a rapidly fatal malignancy and the majority of patients with glioblastoma suffer from a poor quality of life (2,3). Currently, the standard clinical treatment is surgical resection of the malignant tissues, followed by radiotherapy and chemotherapy (4–7). However, patients that receive these treatments may rapidly develop resistance to chemotherapy (8). Recent studies have focused on the identification of candidate biomarkers of glioblastoma development, in order to produce a more effective therapeutic strategy (9–11).

Transcription factors (TFs) have important roles in the transcriptional networks that regulate gene expression, and modify and control cancer phenotypes (12,13). Differentially expressed TFs in glioblastoma, and their downstream gene targets, may be potential therapeutic biomarkers of glioblastoma (12,13). O^6^-methylguanine DNA methyltransferase (MGMT) promoter hypermethylation (14,15) and isocitrate dehydrogenase 1 (16–18) have previously been suggested as potential therapeutic targets, and regulation of MGMT expression has been reported in numerous clinical studies (19,20). It has been suggested that MGMT expression may be regulated by inhibiting its upstream TF, such as SP1 in glioblastoma (21).

Sun *et al* (22) collected mRNA expression data (GSE4290) from patients with brain tumors, and demonstrated that downregulation of stem cell factor (SCF) inhibits tumor-mediated angiogenesis and glioma growth *in vivo*, whereas overexpression of SCF was associated with reduced survival in patients with malignant glioma. Numerous studies have identified glioblastoma-associated genes based on the GSE4290 dataset, with the aim of improving diagnosis of glioma at the molecular level (23,24). However, the importance of differentially expressed TFs has yet to be explored. The present study aimed to identify the differentially expressed TFs in glioblastoma, and the corresponding critical pathways involved in glioblastoma development.

In the present study, the raw mRNA data of Sun *et al* (22) was downloaded from the Gene Expression Omnibus (GEO), and the differentially expressed genes (DEGs) in glioblastoma samples were identified. Functional enrichment analysis of the DEGs was then performed. TFs associated with the glioblastoma gene expression profile were used to construct a regulatory network. The present study may improve understanding regarding the development of glioblastomas. Furthermore, the differentially expressed TFs may be potential biomarkers for the prognosis and therapy of glioblastoma.

## Databases and methods

### Data acquisition

The raw data was downloaded from the GSE4290 dataset (22) deposited in the GEO (http://www.ncbi.nlm.nih.gov/geo/)(25). The dataset included 23 samples from patients with epilepsy, which are considered non-tumor samples, and 77 glioblastoma (grade 4) tumor samples. The platform was GPL570 [HG-U133_Plus_2] Affymetrix Human Genome U133 Plus 2.0 Array.

### Analysis of DEGs

The raw data was initially analyzed using R software (v.3.0.0; http://www.r-project.org/). The chip data was normalized using the Robust Multichip Averaging method (26) in Affy package (http://www.r-project.org/) (27). The DEGs were then identified using the Limma package (http://www.bioconductor.org/packages/release/bioc/html/limma.html) (28) and tested for multi-test correction by Bayes law (29). Genes with P<0.05 and |log_2_fold change (FC)| >1.5 were considered to be DEGs between the tumor and non-tumor groups.

### Functional enrichment analysis

For functional analysis of the selected DEGs, the DEGs were imported into the Database for Annotation, Visualization and Integrated Discovery (http://david.abcc.ncifcrf.gov/) (30), in order to perform a Gene Ontology (GO) functional enrichment analysis and a Kyoto Encyclopedia of Genes and Genomes (KEGG) (31,32) pathway enrichment analysis. GO analysis encompasses three domains: Biological process, cellular components and molecular functions. P<0.05 was considered to indicate significance.

### Weight of regulatory network

Based on the TRANSFAC^®^ (33) database (http://www.gene-regulation.com/pub/databases.html) and the glioblastoma gene expression profile (http://www.ncbi.nlm.nih.gov/geo/query/acc.cgi?acc=GSE4290), TFs identified in the two datasets were selected and used to establish a regulatory network with their target genes. Combined with the gene expression levels, formulae i and ii were used to calculate the average rank correlation coefficient and formula iii was used to calculate the difference value of Spearman coefficients within the regulatory network. The absolute values of the averages of rank correlation coefficient were defined as weight of TF-gene pairs and the absolute value of difference value was defined as weighted coefficient (28).

(i) $$r_{E_{ij}}=\frac{\sum_{k}\left(x_{ik}-{\bar{x}}_{i})(x_{jk}-{\bar{x}}_{j}\right)}{\sqrt{\sum_{k}\left(x_{ik}-{\bar{x}}_{i}\right)\sum_{k}\left(x_{jk}-{\bar{x}}_{j}\right)}}$$

(ii) $$\left|{\bar{r_{E}}}_{ij}\right|=\frac{1}{2}\left|r_{E_{ij1}}+r_{E_{ij2}}\right|$$

(iii) $$\left|\Delta r_{E_{ij}}\right|=\frac{1}{2}\left|r_{E_{ij1}}-r_{E_{ij2}}\right|$$

where E_ij_ is the TF-target gene between TF V_i_ and gene V_j_; k is the kth sample; V_i_ and V_j_ are ranked by their expression levels in the samples respectively, and X_jk_ is the rank of V_i_ in kth sample, X_ik_ is the rank of V_j_ of kth sample; x_i_, x_j_ are the average ranks of V_i_ and V_j_ in the samples, respectively. *^r^E_ij_*_1_ and *^r^E_ij_*_2_ represent the Spearman coefficients of E_ij_ in compared samples respectively. Permutation test was applied to rank the random difference values. TF-gene pairs with a weighted coefficient >90% of the weighted coefficient value were excluded from further analysis (34).

### Screening of sub-networks within the regulatory network

TFs with a degree >15 in the regulatory network were selected and used to establish sub-networks with their target genes. The weight of TF-gene pairs in the sub-networks were scored using the following methods. Initially, the weighted coefficients of all TF-gene pairs within the regulatory network were ranked and defined as a background set (E), whereas the sub-networks were considered as an objective set (S). The score of S enriched into E was then calculated by gene set enrichment analysis (35), according to formula iv:

(iv) $$\begin{array}{l} P_{hit}(S,i)=\sum_{E_{j}\in S,j\leq i}\frac{{\left|r_{j}\right|}^{P}}{N_{R}},\\ where\\ N_{R}=\sum_{E_{j}\in S}{\left|r_{j}\right|}^{P}\\ P_{miss}(S,i)=\sum_{E_{j}\in S,j\leq i}\frac{1}{N-N_{H}} \end{array}$$

where E_j_ is the jth TF-target in the ranked regulatory pairs; r_j_ is the weight of the jth regulatory pair in background set; P is a parameter and set as 1; N is the number of regulatory pairs in E; N_H_ is the number of regulatory pairs in the subnet S. The enrichment score (ES) is the maximum deviation between P_hit_ and P_miss_.

TF-gene pairs without contribution to the ES were excluded from the analysis (34,35). To estimate the significance of ES of the sub-regulatory networks, ES was converted into Z value (34) using formula v.

(v) $$Z_{s}=\frac{ES-\bar{ES}}{S^{\prime }}$$

where ES (bar) is the mean of the random ES set; and S’ is the standard deviation of the random ES set.

### DEGs in the trimmed subnet

Genes from the gene expression profile were defined as a background set (E), whereas genes in the trimmed subnet were defined as an objective set (S). The P-values of the DEGs were ranked and the ES was calculated using formula vi (34).

(vi) $$\begin{array}{l} P_{hit}(Strimmed,i)=\sum_{g_{j}\in S_{trimmed},j\leq i}\frac{{\left|r_{j}\right|}^{P}}{M_{R}},\\ where\\ M_{R}=\sum_{g_{j}\in S_{trimmed}}{\left|r_{j}\right|}^{P}\\ P_{miss}(S_{trimmed},i)=\sum_{g_{j}\in S_{trimmed},j\leq i}\frac{1}{M-M_{H}} \end{array}$$

where g_j_ is the jth gene in the ranked genes; r_j_ is the magnitude of differential expression of the jth gene; P is a parameter and set as 1; M is the number of genes in L; and M_H_ is the number of genes in S_trimmed_.

Genes that did not contribute to the trimmed subnet ES were excluded. The significance of DEGs in the trimmed subnet was calculated by Z value transformation. The final Z-score was calculated using formula vii. The top five trimmed subnets were selected as the candidate regulatory subnets in glioblastoma.

(vii) $$ZS_{combined_{i}}=Z_{s_{i}}^{\prime }+Z_{trimmed_{i}}^{\prime }$$

## Results

### Identification of DEGs and functional enrichment analysis

**Figure 1:**
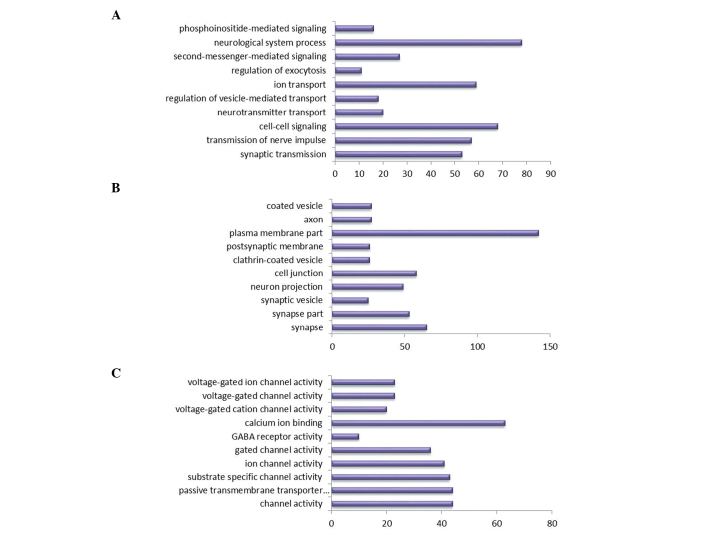
Top 10 GO terms enriched by DEGs. (A) Biological processes; (B) Cellular components; (C) Molecular function. The horizontal axis represents the count of enriched DEGs. The vertical axis represents the different GO terms. GO, gene ontology, DEG, differentially expressed gene; GABA, γ-aminobutyric acid.

**Figure 2:**
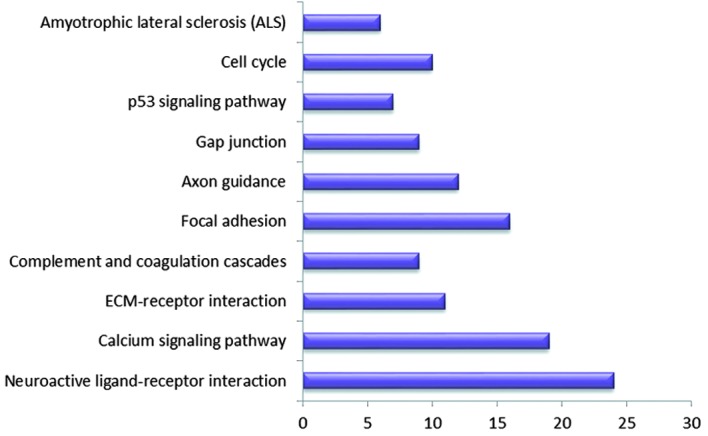
The KEGG pathway enrichment analysis of DEGs. The horizontal axis represents the count of enriched DEGs. The vertical axis represents the different KEGG pathways. KEGG, Kyoto Enclyclopedia of Genes and Genomes; DEG, differentialy expressed gene; ECM, extracellular matrix.

With a cut-off value of P<0.05 and |log_2_FC| >1.5, a total of 676 DEGs were identified, of which 190 were upregulated and 496 were downregulated (Table I). GO analysis demonstrated that the majority of DEGs were enriched in synaptic transmission, regulation of vesicle-mediated transport and ion-gated channel activity (Fig. 1). In addition, KEGG pathway enrichment analysis identified the significantly enriched pathways, which included neuroactive ligand-receptor interaction, calcium signaling pathway, p53 signaling pathway and cell cycle (Fig. 2).

### Establishment of a weighted regulatory network and trimmed subnets

**Figure 3:**
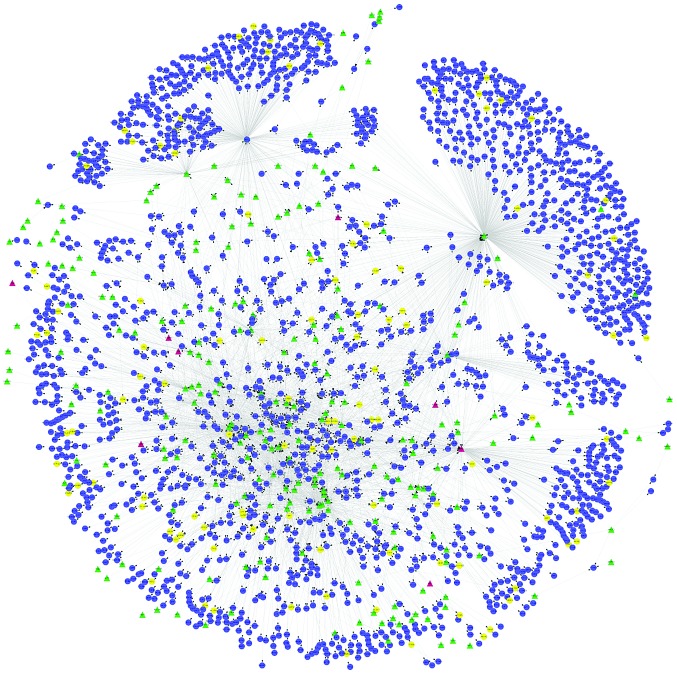
Transcriptional regulation network of genes from the gene expression profile of glioblastomas. The triangles represent differentially expressed transcription factors (TFs) (red, differential expression; green, expression without difference). The yellow circles represent differentially expressed genes (DEGs) regulated by TFs (yellow, DEGs; blue, expression without difference).

To identify TFs in the DEGs, TF-gene pairs were selected based on the TRANSFAC^®^ database and a transcriptional regulatory network (not weighted) with 2,246 nodes and 4,515 regulatory pairs was constructed (Fig. 3). With a weighted coefficient >90% of the random weighted coefficient, 1,312 pairs were excluded by permutation test.

**Figure 4:**
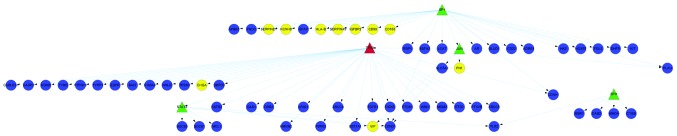
Corresponding sub-regulation network of the transcriptional regulation network. The triangles represent differentially expressed transcription factors (TFs) (red, differential expression; green, expression without difference). The yellow circles represent differentially expressed genes (DEGs) regulated by TFs (yellow, DEGs; blue, expression without difference).

TF-gene pairs of trimmed subnets were calculated and the corresponding DEGs were scored. According to the Z-scores, genes with the top 10 highest Z-scores were identified and the corresponding subnets were constructed (Fig. 4). The candidate TFs and their downstream DEGs are listed in Table II. Only TP53 was identified as a differentially expressed TF in glioblastoma.

## Discussion

In order to identify potential biomarkers for glioblastoma prognosis and therapy, a bioinformatics analysis was performed on the GSE4290 dataset. A total of 676 DEGs were identified, of which 190 were upregulated and 496 were downregulated. The majority of DEGs were functionally enriched in synaptic transmission, regulation of vesicle-mediated transport and ion-gated channel activity. Furthermore, the enriched KEGG pathways of DEGs included neuroactive ligand-receptor interaction, calcium signaling pathway, p53 signaling pathway and cell cycle. Based on the TRANSFAC^®^ database, a transcriptional regulatory network consisting of 2,246 nodes and 4,515 regulatory pairs was constructed. Based on weighted Z-scores, TP53, SP1, JUN, STAT3, and SPI1 were identified as crucial TFs involved in the development of glioblastoma.

As a common malignancy with poor prognosis, glioblastoma tumors harbor various cell types, including vascular cells, microglia, peripheral immune cells and neural precursor cells, which indicates that there is active communication ongoing between the tumor cells and non-tumor cells, and there is a dramatic turnover in the microenvironment (1). It has previously been shown that calcium-mediated transduction systems, together with active gap junctions, have key roles in the communication of GL15 human glioblastoma cells with surrounding cells (36). Eukaryotic cells are capable of using multivesicular bodies for cytoplasmic trafficking and release of exosomes, which may transfer genetic information between non-immune cells (37). The pathway enrichment results of the present study demonstrated that the DEGs in glioblastoma were enriched in ion-gated channels, gap junction signaling, vesicle-mediated transport signaling and cell-cell signaling, supporting the crucial role of calcium transport, coupled with gap junctions, in the invasive capabilities of glioblastoma. Therefore monitoring these signaling pathways may aid prediction of tumor progression.

Among the five TFs identified in the present study to be associated with glioblastoma, TP53 was the only DEG. TP53 encodes p53, a well-known tumor suppressor protein (38). The abnormal expression of p53 leads to failures of cell cycle and apoptosis regulation, as well as cancer development (38). However, few studies have investigated the role of p53 as a TF. Notably, the present study also identified JUN as a candidate biomarker, which is a proto-oncogene that encodes a component of the mitogen-inducible immediate-early TF AP1 and c-Jun, and regulates the cell cycle (39). It has previously been reported that the regulation of JUN in the cell cycle and apoptosis is associated with p53 (40). Furthermore, overexpression of MGMT has previously been shown to accompany an increased recruitment of c-Jun in glioblastoma (20); however, the association between TP53 and JUN in glioblastoma progression has yet to be elucidated. TP53 and JUN may act as potential biomarkers for the prognosis of glioblastoma.

SP1 was also identified as a candidate TF and the majority of its downstream targets were differentially expressed in glioblastoma, thus indicating that SP1 may be critical for the development of glioblastoma. Previous studies (19,20) have targeted the transcriptional activity of SP1 to regulate the expression of MGMT and other genes for glioblastoma therapy. SPI1 is also a putative proto-oncogene associated with tumor progression (41), which encodes a protein that functions in the development of lymphocytes (42). SPI1 may influence the development of glioblastoma through regulation of functional immune cells. SPI1 may also be a potential biomarker or therapeutic target for glioblastoma; however, this requires further confirmatory study.

In conclusion, the present study identified DEGs between glioblastoma and non-tumor samples, and a functional enrichment analysis of the DEGs was performed. According to Z-scores, the candidate TFs: TP53, SP1, JUN, STAT3 and SPI1, and their downstream DEGs, may have important roles in the progression of glioblastoma, and may be potential biomarkers for clinical treatment.

## Figures and Tables

**Table I:** Top 10 up- and downregulated differently expressed genes (DEGs) in glioblastoma tissue samples.

DEG	Log_2_FC	P-value
IGFBP2	3.774858	5.10E-19
TOP2A	3.651993	1.15E-17
COL1A2	3.498576	1.94E-13
PTX3	3.131236	5.99E-11
UHRF1	3.129304	2.42E-21
PBK	3.089627	1.95E-16
CRNDE	3.011189	2.54E-15
COL4A1	2.903048	1.37E-15
SERPINA3	2.830651	1.92E-15
CD163	2.812399	4.82E-14
SST	−4.05134	1.28E-26
MAL2	−4.03901	1.68E-19
VSNL1	−3.93557	8.74E-14
TAC1	−3.83201	1.19E-17
CCK	−3.78346	2.53E-16
SYT1	−3.77802	2.03E-13
SYNPR	−3.72399	1.39E-16
STMN2	−3.67837	3.36E-13
RFPL1S	−3.61819	1.47E-19
FAM19A1	−3.60894	1.93E-22

**Table II:** TFs and their regulated-DEGs.

TF	Regulated-DEG	Z score
TP53^a^	CHGA	1.98
SP1	IGFBP2, SERPINA3, CD163, CD99, KCNH8, SERPINE1, HLA-B	1.25
JUN	TP53, VIP, FN1	1.13
STAT3	VIP	1.19
SPI1		1.30

a TP53 is a differentially expressed TF.

TF, transcription factor; DEG, differentially expressed gene.
